# Extirpation of the Uterus

**Published:** 1828-11

**Authors:** 


### EXTIRPATION OF THE UTERUS.

In May 1827, Mr. John M. Banner, surgeon to the North Dispensary, Liverpool, was first called to Mrs. J. on account of retention of urine. The same symptom recurring, he examined the os uteri, and found it painful, thickened, hard, and irregular. On inquiry, it appeared that she had suffered occasional shooting pains from the pubes to the sacrum, for near two years; that these had then become more frequent, accompanied with pain across the loins, sense of weight within the pelvis, and bearing down. The catamenia were irregular. Mrs. J. was forty-four years old, had enjoyed good health till within the last four years, was married at the age of twenty-one, and had two children. In a few years her husband died, and since then she had led an irregular life. She stated that her father died of a cancerous disease of the breast; that it was extirpated twice, and subsequently once from the axilla; that at length he died, after suffering severely for several years.

The removal of the neck of the uterus was proposed at this time, but not assented to.

In July 1828, Mr. B. was again requested to visit her. Various remedies had been used under the care of a physician, with no permanent benefit. Frequent hemorrhages, to a greater or less extent, had taken place; the pains were increased; and a quantity of bloody offensive matter had passed some weeks previously per vaginam.

On examination, he found ulceration to a small extent on one side of the os uteri, and the general health was evidently impaired. She determined to undergo the operation; which, however, Mr. B. thought would be unjustifiable, as no boundary to the disease could be perceived by the most careful examination, the hardness of the neck appearing to extend to the body of the uterus, as far as could be felt. In this state she continued until the beginning of August, when, in consequence of the case which had occurred to Dr. Blundell, it was resolved to operate.

The patient being placed on her back, as for the operation of lithotomy, but without tying the hands or feet, Weiss's speculum vaginæ was introduced, and held by an assistant; a strong hook was passed into the anterior part of the cervix, and the uterus drawn down, with little difficulty or pain, to about half an inch from the os externum. A strong aneurism needle, with a handle, having its extremity pointed, and armed with a double ligature, was then passed through the neck of the uterus, the hook withdrawn, and the ligature held by an assistant, whilst the speculum was also removed, and the labia held out of the way by those on each side. A semicircular incision was then made on the inferior part of the cervix, through the vagina and peritoneum, and widened with a hernia knife from one broad ligament to the other. Afterwards a similar incision was made at the superior part, and extended as before, so that the broad ligaments and fallopian tubes only remained to be divided: to accomplish this, the index finger of the left hand was passed through the upper opening, and the middle through the lower, including the right broad ligament between them: an incision was then made with a scalpel between the fingers and uterus, close to its body. The nearest part of the included portion was thus divided, and was attended with slight hemorrhage. Some time was lost in endeavouring to secure the bleeding vessel, which however proved unsuccessful. The hemorrhage not being very profuse, the operation proceeded; but, finding the former plan tedious and difficult, the fundus was brought down by passing two fingers through the upper opening, and then the strong hook between the hand and uterus, the point of which was easily pressed into the fundus, and thus the object was quickly accomplished. The fallopian tubes and remaining part of the broad ligaments were now distinctly seen, and, by passing the fingers beneath them, were divided with the common scalpel, close to the uterus. This was by far the most painful part of the whole.

During the operation, (which lasted twenty-five minutes,) the patient was troubled with retching. About six ounces of blood were lost. The intestines did not protrude, nor interfere with any part of the process. Immediately after, the patient was as well as could be expected, and she was put into bed. In the course of twenty minutes or half an hour, she vomited severely, and became very faint; a coagulum of about eight ounces was expelled. Vinegar and water were applied to the abdomen and upper part of the thighs: she then rallied, and after some time complained of pain at the lower part of the abdomen; and, the vomiting recurring, another coagulum, rather larger than the first, came away. She was now in a state of syncope; the retching remained severe, and almost incessant. One hundred drops of tincture of opium were given, but immediately rejected. Small quantities of brandy were administered; the cold cloths continued; and the patient kept in the horizontal position. The hemorrhage did not return after the expulsion of the second coagulum, and the pain in the abdomen subsided. She again rallied a little; and in the evening, as the vomiting continued extremely distressing, two grains of opium were given. The patient was relieved for two hours; the symptoms then returned, and four grains were given, which gave relief for the same length of time as the first dose.

September 3d.—Passed a very restless night. Countenance pale and dejected; pulse ninety-six, and weak; skin moist, and of a natural temperature; slight pain in the abdomen and back; vomiting less frequent. Has not passed any urine since the operation, nor had any evacuation from the rectum. The catheter was introduced, and ten ounces of high-coloured urine withdrawn.

Vespere.—Bowels purged freely by injections, and small doses of the sulphate of magnesia in infusion of roses. Vomiting and pain relieved.

4th.—General appearance as yesterday; pain in abdomen slightly increased on pressure; little or no tension; pulse ninety-four, rather fuller; vomiting much the same; tongue slightly furred; complains of great thirst; bowels freely open. At noon, the pulse had risen to 108, and twenty-four leeches were applied to the abdomen. Passed urine twice.

Vespere.—Pain little abated; pulse remains quick, and rather hard. V.S. to syncope. Twelve ounces were taken away.

She lived till the 6th, the symptoms gradually increasing till six A.M., when she died.

*The appearance of the uterus.*—The uterus was much larger than in the healthy state; several tubercles of various sizes were loosely attached to the body and fundus: they were round and very hard. The cervix and body were considerably thicker and harder than natural. Ulceration had taken place on the os uteri, particularly at the lower lip: a section exhibited the common appearance of scirrhus; a circumscribed hardness was very perceptible, extending from the cervix to the body on the left side. Several small, round, hard tumors were imbedded in the substance of the fundus.

*Examination*, five hours after death.—No tension of the abdomen. On examining the cavity, the omentum and intestines were found highly inflamed, and adherent to each other by an effusion of lymph. Several folds of small intestine filled the pelvis, and were more inflamed and adherent than those above. The lowest convolutions were firmly adhering to the cut surfaces made in the operation and to each other, so as completely to close the aperture from within; and only a small quantity of serum was effused. The bladder remained in sitû.

The peritoneum lining the pelvis had in general a greenish and somewhat dull appearance, which by some present was thought to be of a gangrenous character; but its texture was perfectly firm and unyielding. The ovaria were retained in their usual position by the remainder of the round and broad ligaments. The fimbriated extremity of the left fallopian tube was found closed and distended with serum nearly to the size of a hen's egg, and gradually narrowing along an inch of the tube to a point, where it was again closed. The ovaria were as usual in persons who had borne children, being flattened and corrugated, as if covered with cicatrices. The duplicatures of peritoneum forming the broad ligaments were more separated below than above, where they inclose the ovaria, and were thus kept in union. A very careful examination was made to discover, if possible, the source of the hemorrhage; the arteries were probably retracted, as none divided could be found; but the mouths of several considerable veins were seen distinctly on the right side, where the layers of the broad ligament were separated, and traced to the plexus at the side of the pelvis. The branches of the internal iliac on this side, and the spermatic arteries, were examined, but no irregularity as to size or distribution was discovered.

